# Neonatal mortality in the neonatal intensive care unit of Debre Markos referral hospital, Northwest Ethiopia: a prospective cohort study

**DOI:** 10.1186/s12887-020-1963-z

**Published:** 2020-02-15

**Authors:** Animut Alebel, Fasil Wagnew, Pammla Petrucka, Cheru Tesema, Nurilign Abebe Moges, Daniel Bekele Ketema, lieltework Yismaw, Mamaru Wubale Melkamu, Yitbarek Tenaw Hibstie, Belisty Temesgen, Zebenay Workneh Bitew, Animen Ayehu Tadesse, Getiye Dejenu Kibret

**Affiliations:** 1 College of Health Science, Debre Markos University, P.O. Box 269, Debre Markos, Ethiopia; 2 Faculty of Health, University of Technology Sydney, Ultimo, NSW Australia; 3 College of Nursing, University of Saskatchewan, Saskatoon, Canada; 4 School of Life Sciences and Bioengineering, Nelson Mandela African Institute of Science and Technology, Arusha, Tanzania; 5 Debre Markos Referral Hospital, Debre Markos, Ethiopia; 6 Department of Nursing, St. Paul’s Hospital Millennium Medical College, Addis Ababa, Ethiopia; 7 Department of Medical Parasitology, College of Medicine and Health Sciences, Bahir Dar University, Bahir Dar, Ethiopia

**Keywords:** Neonatal mortality, Neonatal intensive care unit, Debre Markos referral hospital, Northwest Ethiopia

## Abstract

**Background:**

Neonatal mortality remains a serious global public health problem, but Sub-Saharan Africa (SSA), in particular, is largely affected. Current evidence on neonatal mortality is essential to inform programs and policies, yet there is a scarcity of information concerning neonatal mortality in our study area. Therefore, we conducted this prospective cohort study to determine the incidence and predictors of neonatal mortality at Debre Markos Referral Hospital, Northwest Ethiopia.

**Methods:**

This institutionally-based prospective cohort study was undertaken among 513 neonates admitted to the neonatal intensive care unit of Debre Markos Referral Hospital between December 1st, 2017 and May 30th, 2018. All newborns consecutively admitted to the neonatal intensive care unit during the study period were included. An interviewer administered a questionnaire with the respective mothers. Data were entered using Epi-data™ Version 3.1 and analyzed using STATA™ Version 14. The neonatal survival time was estimated using the Kaplan-Meier survival curve, and the survival time between different categorical variables were compared using the log rank test. Both bi-variable and multivariable Cox-proportional hazard regression models were fitted to identify independent predictors of neonatal mortality.

**Results:**

Among a cohort of 513 neonates at Debre Markos Referral Hospital, 109 (21.3%) died during the follow-up time. The overall neonatal mortality rate was 25.8 deaths per 1, 000 neonate-days (95% CI: 21.4, 31.1). In this study, most (83.5%) of the neonatal deaths occurred in the early phase of neonatal period (< 7 days post-partum). Using the multivariable Cox-regression analysis, being unemployed (AHR: 1.6**,** 95% CI: 1.01, 2.6), not attending ANC (AHR: 1.9, 95% CI: 1.01, 3.5), not initiating exclusive breastfeeding (AHR: 1.7, 95% CI: 1.02, 2.7), neonatal admission due to respiratory distress syndrome (AHR: 2.0, 95% CI: 1.3, 3.1), and first minute Apgar score classification of severe (AHR: 2.1, 95% CI: 1.1, 3.9) significantly increased the risk of neonatal mortality.

**Conclusion:**

In this study, we found a high rate of early neonatal mortality. Factors significantly linked with increased risk of neonatal mortality included: unemployed mothers, not attending ANC, not initiating exclusive breastfeeding, neonates admitted due to respiratory distress syndrome, and first minute Apgar score classified as severe.

## Background

Neonatal mortality (NM) is defined as the death of neonates within the first 4 weeks of life (i.e., the neonatal period) [1]. Early neonatal mortality (ENM) refers to the death of neonates within the first week of life [2] and late neonatal mortality (LNM) refers to the death of neonates from the seventh day until the 28th day post-birth [3]. NM remains a serious public health challenge throughout the world, most notably in low and middle income countries (LMICs). In 2015, an estimated 2.7 million neonatal deaths occurred worldwide, accounting for 45% of deaths in under-5 years of age children [4, 5]. Almost all (99%) newborn deaths occurred in LMICs, with the least progress in reducing neonatal deaths being achieved in Africa and South Asia [2].

Furthermore, this trend is amplified in Sub-Saharan Africa (SSA) which carries the highest NM incidence in the world, and shows the lowest progress in reducing NM [6]. Ethiopia was listed as having the third highest number of neonatal deaths in eastern SSA in 2013 [7]. According to the recent Ethiopian Demographic and Health Survey (EDHS, 2016) report, NM in Ethiopia was reported as 29 deaths per 1000 live births [8].

Evidence suggests that about 75% of the neonatal deaths in low and middle income countries (LMICs) are preventable with existing simple and low cost tools, such as antibiotics for pneumonia and sepsis, sterile blades to cut the umbilical cords, and knit caps and kangaroo care to keep babies warm [9, 10]. As an intervention, in 2005, the United Nations launched MDG #4, which aimed to reduce the mortality rate of under-five children by 66% by the end of 2015 [4]. Despite NM reduction from 5.1 million in 1990 to 2.7 million in 2015, the global decline in NM between 1990 to 2015 was slower than post-neonatal under-five mortality (1–59 months) (i.e., 47%, compared with 58%) [4].

A few studies conducted in Ethiopia found NM incidence in Ethiopia ranged from 1.3 per 1000 live births in Oromia Region [11] to 62.5 per 1000 live births in Tigray Region [12]. These studies documented many maternal and neonatal factors significantly associated with NM. Accordingly, maternal education [13], sex of the neonate [14], antenatal care follow-up [14–16], postnatal care follow-up [14], complications during pregnancy [12, 17], birth weight (low birth weight) [15, 16, 18–21], and short birth interval (less than 24 months) [15, 18] were some of the factors significantly associated with NM.

In Ethiopian, the government adapted and implemented different strategies to achieve MDG #4. For example, Emergency Obstetric and Newborn Care has been adapted to improve neonatal and maternal outcomes, yielding marked success in the reduction of under-five mortality [22, 23]. There was variation in the reduction of mortality among different childhood age groups, with the least reduction being achieved in the neonatal groups compared to infant and child groups [24]. Even though different interventions have been made by the government, NM in Ethiopia remained high. Therefore, this prospective cohort study was designed to identify the risk factors of NM [15]. Results obtained from this study will inform planning interventions to improve the survival of neonates in the study area, and similar settings of Ethiopia. Additionally, this study will serve as baseline information for further studies.

### Objectives

- To determine the incidence of mortality among neonates admitted to the neonatal intensive care unit of Debre Markos Referral Hospital
- To identify predictors of mortality among neonates admitted to the neonatal intensive care unit of Debre Markos Referral Hospital

## Methods

### Study design and setting

This institutionally-based prospective cohort study was undertaken between December 1st, 2017 and May 30th, 2018 in the Neonatal Intensive Care Unit (NICU) of the Debre Markos Referral Hospital. Debre Markos town is located 300 km from Addis Ababa, the capital city of Ethiopia, and 256 km from Bahir-Dar, the capital of Amhara Regional State. Debre Markos Referral Hospital is the only referral hospital found in East Gojjam Zone. This hospital serves more than 3.5 million people in its catchment area. As well, the hospital provides neonatal intensive care services for critically ill neonates and those who needs neonatal care. The NICU has 27 nurses, one pediatrician, and two general practitioners. Besides, the unit has 10 NICU beds, four kangaroo mother care beds, 19 mother side beds, eight radiant warmers, and six incubators. The common nursing procedures in the NICU are: phototherapy, umbilical transfusion, oxygen administration, nasogastric tube insertion, intravenous infusion, urinary catheterization, lumbar puncture, and Continuous Positive Airway Pressure Ventilation (CPAP). In 2017, this hospital provided neonatal intensive care services for 1419 neonates.

### Population

The source and study population for this study were all neonates admitted to the NICU of Debre Markos Referral Hospital from the period of December 1st, 2017 to May 30th, 2018. We excluded neonates whose mothers were unable to communicate due to serious illness, neonates admitted without mothers, and mothers with psychiatric illnesses.

### Sample size determination and sampling procedure

We included all newborns consecutively admitted to the NICU of Debre Markos Referral Hospital during the study period. Even though we included all neonates consecutively admitted to the NICU, we checked the adequacy of the sample size based on our objectives. Accordingly, for the first objective, the minimum required sample size was calculated using a single population proportion formula by considering the following statistical assumptions: *P* = proportion (22%) of NM, which was obtained from a study conducted in Tigray region [12], Z α/2 = corresponding Z score of 95% CI and d = margin of error (5%). In our study, *N* = (1.96)^2^ *0.22*0.78/ (0.05)^2^ = 264 neonates. Finally, after assuming a 10% loss to follow-up, the final sample size required for the first objective was 291neonates.

For the second objective, the adequacy of sample size was checked using a sample size calculation for the survival analysis formula by considering not initiating exclusive breastfeeding and having neonatal complications as the major predictor variables using STATA™ Version 14 statistical software (Table 1). This calculation yielded a sample size requirement of 146 neonates. Finally, we included a total of 513 neonates admitted to the NICU of Debre Markos Referral Hospital from December 1st, 2017 and May 30th, 2018. Regarding sampling technique, we used a consecutive sampling technique and followed the neonates for the 28 day post-birth period. When the neonates were discharged before 28 days, weekly phone calls were undertaken to monitor the newborn outcomes at home.

**Table 1:** Sample size calculation to assess the incidence and predictors of neonatal mortality at Debre Markos Referral Hospital, Northwest Ethiopia, 2018

Variables	Assumptions	Hazard ratio	Total sample size	10% loss to follow-up
Not initiating exclusive breastfeeding	Power = 80% CI = 95 *π*_1_= *π*_2_ = ½	7.5	125	138 [12]
Having neonatal complications	Power = 80% CI = 95 *π*_1_= *π*_2_ = ½	7.1	132	146 [12]

Note: ***π***_**1**_ **and** ***π***_**2**_***:*** the proportions to be allocated between exposed and non-exposed groups HR: hazard ratio

### Variables of the study

The dependent variable for this study was the time to death. The independent variables were: sociodemographic characteristics including age of the neonate, sex of the neonate, marital status of the mother, residence, age at first marriage, age at first birth, educational status of the mother, and occupation of the mother; maternal obstetric and health service related information including exclusive breastfeeding, maternal complications at birth, antenatal care follow-up, abortion history, medical disease(s) during pregnancy, and distance from a health facility; and neonatal related predictors including gestational age, neonatal complications, birth weight, birth type, birth interval, weight for gestational age, birth defect, Apgar 1-min score, Apgar 5-min score, and neonatal resuscitation.

### Operational definitions

#### Event

Death of a neonate at specific time (day) within the 28 days of follow-up as evidenced by physician confirmation or telephone verification from mothers [1].

Neonatal mortality was calculated by dividing the number of neonates died during the study period to the neonate-days [25]. This is the appropriate measurement for our study because, since this study included only sick neonates admitted to the NICU, calculating the NM per 1000 live births could overestimate the NM.

#### Early neonatal mortality

Probability of death before seven completed days of life [2].

#### Late neonatal mortality rate

Probability of dying between seven completed days and before 28 completed days of life [3].

#### Censored

Neonates who were alive at the end of follow up, and/or lost-to-follow-up.

### Data collection procedures and quality control

We used an interviewer administered pre-tested and structured questionnaire to collect the data. The questionnaire was prepared from relevant literature and the WHO standard verbal autopsy questionnaire [26]. To maintain data quality, the questionnaire was initially developed in English then translated to Amharic and back to English. The tool was pre-tested on 5% of the total sample size at Finote Selam Hospital. The data from this piloting of the tool was not analyzed or reported in this study.

All data were collected at the time of admission through interviewing all mothers whose neonates were admitted to the NICU of Debre Markos Referral Hospital. In addition, clinical data were obtained by assessment of the neonates and mothers at the time of admission. The neonates were followed for a maximum of 28 days, using two alternative strategies. The data collector visited the neonate daily, while in the hospital. After the neonate was discharged, the data collector contacted the mother every 7 days via a telephone call and inquired about the neonate’s condition and survival status. When death occurred, the date and cause of death was recorded. If the neonate died in the hospital, the cause of death was confirmed by physician, however, if the neonate died at home after discharged from the hospital, it was assessed by provider judgement when the newborn was still in the NICU.

All bachelor’s degree prepared nurses currently working in the NICU of Debre Markos Referral Hospital were involved as data collectors. To assure data quality, daily supervision was done by principal investigators and the NICU supervisor (who was a BSc prepared nurse). A one-day training session was given for both data collectors and the supervisor concerning the data collection tool and data collection process. Moreover, all collected data were examined for completeness and consistency during the data management, storage, and analysis phase by the research team members.

### Statistical analysis

We used Epi-data™ Version 3.1 for data entry and STATA™ Version 14 statistical software for data analysis. Before analysis, data were cleaned and edited. The necessary assumption of Cox-proportional hazard regression model was checked using the Schoenfeld residual test and the Log-Log plot. The neonate cohort characteristics of continuous data were described in terms of central tendency (mean or median), dispersion (standard deviation or inter quartile range) and in the frequency distribution for categorical data. Finally, the outcomes of neonates were dichotomized into censored or death categories. The Kaplan Meier survival curve was used to estimate survival time, and log rank test was used to compare the survival curves. Bi-variable Cox-proportional hazards regression model was fitted for each explanatory variable. Moreover, those variables having *p*-value ≤0.25 in bivariate analysis were fit into the multivariable Cox-proportional hazard regression model. Hazard ratio with 95% confidence interval and *p*-values were used to measure the strength of association and to identify statistically significant predictors. In the multivariable analysis, variables having *P*-value < 0.05 were considered as significant predictors of mortality.

## Results

### Sociodemographic characteristics of the study participants

In this prospective cohort study, a total of 513 neonates consecutively admitted to the NICU of Debre Markos Referral Hospital from the period of December 1st, 2017 to May 30th, 2018 were included. The response rate of this study was 100% with 59.5% of the neonates being females and about half (51.1%) being from urban areas. The majority (95.1%) of the study participants were from Orthodox religions. The mean age of the neonates at the time of admission was 3.9 days (SD ± 0.2); with the mean age of mothers at first marriage and at first birth reported as 19.3 years (SD ± 0.2) and 23 years (SD ± 0.2) respectively. Regarding marital status, the majority (94.5%) of the mothers were married; nearly, one third (29.8%) completed primary education, and about two-thirds (70%) were unemployed (Table 2).

**Table 2:** Sociodemographic characteristics of the mothers and neonates at Debre Markos Referral Hospital, Northwest Ethiopia, 2018

Variables	Frequency (N)	Percentage (%)
**Sex of the neonate**		
Male	305	59.5
Female	208	40.5
**Neonatal age at admission**		
≤ 3 days	343	66.9
> 3 days	170	33.1
**Residence**		
Urban	251	48.9
Rural	262	51.1
**Age at first marriage**		
< 18 years	242	47.2
≥ 18 years	271	52.8
**Age at first birth**		
< 20 years	99	19.3
20–24 years	239	46.6
> 24 years	175	34.1
**Religion**		
Orthodox	488	95.1
Other	25	4.9
**Marital status**		
Married	485	94.5
Other	28	5.5
**Maternal educational status**		
Unable to read and write	191	37.2
Primary	153	29.8
Secondary	78	15.2
Tertiary	91	17.7
**Maternal occupation**		
Employed	154	30.0
Unemployed	359	70.0

### Maternal obstetric and health service-related characteristics

More than half (63.2%) of the mothers were RH positive. The majority (94.7%) of the mothers had ANC follow-up during the current pregnancy. Almost one-third (30.1%) experienced complications during delivery, with 5.5% (27) reporting having an illness during the current pregnancy. Among the 27 individuals reporting a disease, 12 (44%) of the mothers had HIV infection. During the current pregnancy, the majority (84.2%) of the mothers received the tetanus toxoid vaccine. With respect to mode of delivery, more than half (63.9%) of the neonates were delivered vaginally, and 86.4% were exclusively breastfed. It is noted that more than half (63.8%) of the mothers lived < 5 km from a health facility (Table 3).

**Table 3:** Maternal and health service-related characteristics of the study participants at Debre Markos Referral Hospital, Northwest Ethiopia, 2018

Variables	Frequency (N)	Percentage (%)
**Number of children**		
Primipara	248	48.3
2–4 children	219	42.7
≥ 5 children	46	9.0
**Gravidity**		
< 2 pregnancies	228	44.4
≥ 2 pregnancies	285	55.6
**RH immunization status**		
Positive	324	63.2
Negative	102	19.9
Unknown	87	16,9
**Complication(s) during labor**		
Yes	159	30.1
No	354	69.0
**Antenatal Care follow-up**		
Yes	486	94.7
No	27	5.3
**Number of Antenatal Care follow-ups (486)**		
< 4	170	35.0
≥ 4	316	65.0
**History of abortion**		
Yes	78	15.2
No	435	84.8
**Types of abortions (78)**		
Spontaneous	73	93.6
Induced	5	6.4
**History of stillbirth**		
Yes	41	8.0
No	472	92.0
**Number of stillbirths** (41)		
< 2	34	82.9
≥ 2	7	17.1
**Pregnancy related complications**		
Yes	73	14.2
No	440	18.8
**Types of complications (73)**		
Hypertension	44	60.3
Hemorrhage	10	13.7
Others	20	27.4
**Any disease during current pregnancy**		
Yes	27	5.3
No	486	94.7
**Types of diseases (27)**		
HIV	12	44.4%
Malaria	6	22.2
Other	9	33.3
**TT vaccine**		
Yes	432	84.2
No	81	15.8
**Number of TT vaccines (432)**		
One	74	17.1
≥ Two	358	82.9
**Mortality of sibling**		
Yes	63	12.3
No	450	87.7
**Mode of delivery**		
Vaginal	328	63.9
Caesarean Section	104	20.3
Instrument	81	15.8
**Exclusive breastfeeding**		
Yes	443	86.4
No	70	13.6
**Distance from health institution**		
< 5 km	326	63.6
5–10 km	91	17.7
> 10 km	96	18.7

### Neonatal related characteristics

Among all neonates admitted to the NICU of Debre Markos Referral Hospital, about half (53%) were low birth weight. One-third (33.5%) of the neonates admitted to the NICU were preterm. More than three-fourths (76.2%) of the neonates received immediate newborn care, with 60.6% not requiring resuscitation (Table 4).

**Table 4:** Neonatal related characteristics of study participants at Debre Markos Referral Hospital, Northwest Ethiopia, 2018

Variables	Frequency(N)	Percentage (%)
**Birth weight**		
Normal (≥2500 g)	272	53.0
Low birth weight (< 2500 g)	241	47.0
**Gestational age**		
Preterm	172	33.5
Term	330	64.3
Post term	11	2.1
**Weight for gestational age**		
Appropriate	404	78.7
Small for Gestational Age	99	19.3
Large for Gestational Age	10	2.0
**Birth type**		
Single	423	82.5
Multiple	90	17.5
**Birth interval**		
Not applicable	239	46.6
< 2 years	77	15.0
≥ 2 years	187	38.4
**First minute Apgar score**		
Severe	29	5.7
Other than severe	484	94.3
**Fifth minute Apgar score**		
Severe	30	3.8
Not severe	383	94.2
**Immediate newborn care**		
Yes	391	76.2
No	60	11.7
Unknown	62	12.1
**Resuscitation**		
Yes	147	28.6
No	316	60.6
Unknown	50	9.8
**Neonate inborn or out born**		
**Inborn**	335	65.3
**Out born**	178	34.7

### Incidence of neonatal mortality

**Fig. 1:**
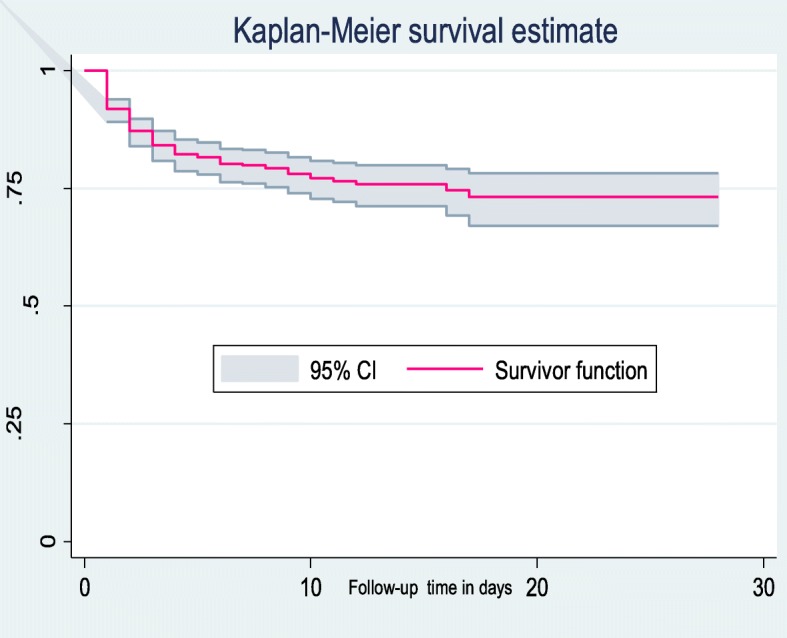
The overall Kaplan-Meier survival curve with 95% confidence interval showing the survival time of neonates at Debre-Markos Referral Hospital, Northwest Ethiopia

**Fig. 2:**
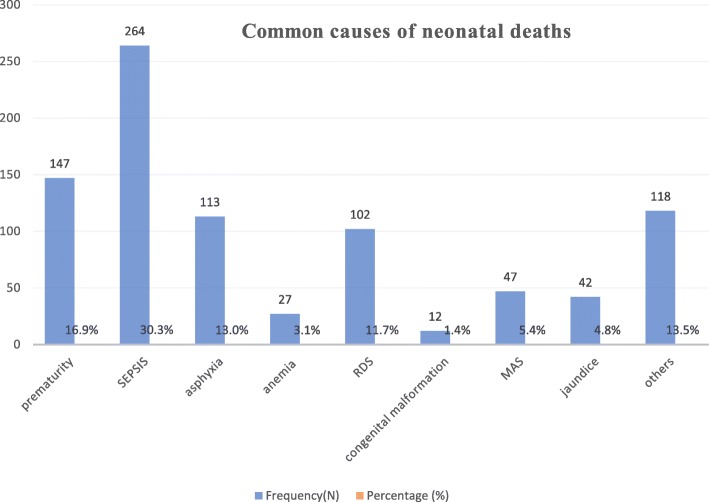
The common causes of neonatal deaths at Debre Markos Referral Hospital from December 1st, 2017 to May 30th, 2018

The neonates were followed for a minimum of 1 h to a maximum of 28 days. The cohort contributed a total of 4223 neonate-days. The median follow-up time of the entire cohort was found to be 7 days (IQR: 4–11 days). During the time of follow up time, about 21.3% of the neonates died. However, most (83.5%) of the neonatal deaths occurred in the early phase of neonatal period (< 7 days). The overall mortality rate (incidence density) of this cohort was found to be 25.8 deaths per 1000 neonate-days (95% CI: 21.4, 31.1). In this study, we observed a high early NM [ENM] (39.4 (95% CI: 32.3, 48.1) per 1000 neonate-days) as compared to late NM [LNM] (6.8 (95%CI: 3.9, 12.0) per 1000 neonate-days) (Fig. 1). Regarding the cause of neonatal deaths, about one-third (33.5%) of neonatal deaths were attributed to preterm and slight less (30.6%) of the neonatal deaths were attributed to neonatal sepsis (Fig. 2).

### Predictors of neonatal mortality

In the bi-variable Cox-regression analysis, significant predictors (*p*-value ≤0.25) of NM included: age of the neonate, admitted due to respiratory distress syndrome, admitted due to prematurity, birth interval, low first minute Apgar, number of children, mother’s occupation, age at first marriage, ANC follow-up, and exclusive breastfeeding practice. In the final model, only five variables were identified as significant predictors of NM. Accordingly, neonates delivered by unemployed mothers were 1.6 (AHR: 1.6**,** 95% CI: 1.01, 2.6) times more likely to die as compared to employed mothers.

Neonates born to mothers who had not attend ANC follow-up were 1.9 times (AHR: 1.9, 95% CI: 1.01, 3.5) more likely to die as compared to neonates born from mothers who had ANC follow-up. Moreover, neonates who did not exclusively breastfed were 1.7 times (AHR: 1.5, 95% CI1.02, 2.7) more likely to die as compared to exclusively breastfed neonates. Furthermore, neonates who were admitted due to respiratory distress syndrome were 2.0 times (AHR: 2.0, 95% CI: 1.3, 3.1) more likely to die as compared to neonates admitted due to other cases. Finally, this study found that neonates whose first minute Apgar scores were classified as severe were 2.1 times (AHR: 2.1, 95% CI: 1.1, 3.9) more likely to die as compared to those neonates whose first minute Apgar scores were classified as not severe (Table 5).

**Table 5:** The bi-variable and multivariable Cox-regression analysis of predictors of neonatal mortality at Debre Markos Referral Hospital, Northwest Ethiopia, 2018

Variables	Survival status		CHR (95%CI)	AHR (95%CI)
	Event	Censored		
**Neonatal age at admission**				
≤ 3 days	82	261	1	1
> 3 days	27	143	0.6 (0.4, 1.00)	0.8 (0.5, 1.3)
**Age at first birth**				
< 20 years	19	80	0.8 (0.6, 1.5)	1.1 (0.6, 1.9)
20–24 years	46	193	1	1
> 24 years	44	131	1.3 (0.9, 2.0)	1.4 (0.9, 2.2)
**Maternal occupation**				
Employed	25	129	1	1
**Unemployed**	**84**	**275**	**1.4 (0.9, 2.3)**	**1.6 (1.01, 2.6)**^a^
**Number of children**				
Primipara	57	191	1	1
2–4 children	44	175	0.8 (0.5, 1.2)	0.7 (0.4, 1.3)
≥ 5 children	8	38	0.7 (0.3, 1.4)	0.6 (0.2, 1.4)
**Antenatal care follow-up**				
Yes	96	390	1	1
**No**	**13**	**14**	**2.6 (1.5, 4.6)**	**1.9 (1.01, 3.5)**^a^
**Exclusive breastfeeding**				
Yes	84	359	1	1
**No**	**25**	**45**	**2.0 (1.3, 3.2)**	**1.7 (1.02, 2.7)**^a^
**Respiratory Distress Syndrome**				
**Yes**	**41**	**61**	**2.5 (1.7, 3.7)**	**2.0 (1.3, 3.1)**^a^
No	68	343	1	1
**Premature Birth**				
Yes	42	105	1.5 (1.0, 2.1)	1.1 (0.7, 1.7)
No	67	299	1	1
**Birth interval**				
Not applicable	56	183	1.1 (0.6, 1.8)	0.9 (0.5, 1.6)
< 2 years	18	59	1	1
≥ 2 years	35	162	0.7 (0.4, 1.3)	0.8 (0.5, 1.5)
**First minute Apgar score**				
**Severe**	**13**	**16**	**2.7 (1.5, 4.8)**	**2.1 (1.1, 3.9)**^a^
Not severe	96	388	1	1

^a^**Significant predictors in the multivariable analysis**

## Discussion

Despite numerous innovations and interventions made to improve the survival of newborns, NM remains a serious global public health concern, notably in LMICs. Therefore, we conducted this prospective cohort study to determine NM at Debre Markos Referral Hospital, Ethiopia. Accordingly, the overall NM at Debre Markos Referral Hospital was found to be 25.8 per 1000 neonate-days (95% CI: 21.4, 31.1). This finding aligns with previous studies conducted in eastern Ethiopia reporting NM incidence of 28.37 per 1000 live births [27], a study at Wolaita Sodo University Teaching and Referral Hospital reporting 27 per 1000 neonates-days [28], and the EDHS (2016) which reported 29 per 1000 live births [8].

However, our finding is lower than the results of a number of studies, such as one conducted in Northern Ethiopia (62.5 per 1000 live births) [12], Jimma Zone Southwest Ethiopia (35.5 per 1000 live births) [25], Nigeria (41 per 1000 live births) [15], and Burkina Faso (46.5 per 1000 live births) [29]. Conversely, our finding is much higher than NM incidence reported in Butajira District, South Central Ethiopia (1.3 per 1000 live births) [11]. The above variations between studies could be explained, in part, by the differences in sample size, study settings, follow-up period, and socio-demographic characteristics of study participants.

In this study, we observed a high ENM (39.4 deaths per 1000 live births) as compared to LNM (6.8 per 1000 live births). This mortality difference is consistent with studies reported from Northern Ethiopia [12], Butajira District, South Central Ethiopia [11], and Jimma Zone Southwest Ethiopia [25]. This mortality difference between the groups could be attributed to the fact that most of the neonatal deaths in resource limited settings are associated with delivery, intrapartum, and the immediate newborn care practices. Besides, in our study, more than half (61.2%) of the neonatal deaths are attributed to birth asphyxia, neonatal sepsis, and prematurity. From this finding we can conclude that more neonatal survival interventions should be targeted towards the intra-partum period as well as in immediate and early neonatal periods. This finding aligns with the World Health Organization report, which shows up to half of all deaths occur in the first 24 h of life, and 75% occur in the first week, with the 48 h immediately following birth cited as the most crucial time for newborn survival [30].

In this cohort study, maternal occupation was significantly associated with NM. Accordingly, neonates delivered by unemployed mothers were more likely to die as compared to their employed counterparts. This finding contradicts findings from a study reported from India, which shows the odds of neonatal death were lower among infants born from unemployed mothers than employed mothers [31]. The possible explanation for these contradictory findings might be due to the difference in socioeconomic and sociodemographic status of the mothers, as employed mothers are more educated and have a better income than unemployed mothers. Other evidence suggested that NM was significantly associated with maternal educational status and income [32].

The current study found that a lack of ANC follow-up was significantly associated with increased NM. Neonates born to mothers who did not participate in ANC follow-up were at higher risk of death as compared to neonates born to those who had undertaken ANC follow-up. This finding aligns with previous studies conducted in Ethiopia as well as in other SSA countries [14–16]. ANC visits may help to reinforce maternal education and compliance, and provide an opportunity for screening for warning signs of pregnancy complications and treatment of infections [33]. In addition, ANC provides an excellent opportunity for health care workers to teach mothers how to recognize warning signs of complications during pregnancy, labor, and delivery whilst encouraging them to plan clean and safe deliveries preferably with trained assistants [34, 35]. At the time of ANC follow-up health care providers can provide information regarding postpartum care, newborn care, breastfeeding, pregnancy risk signs, and appropriate actions to be taken [36].

Additionally, exclusive breastfeeding practice was significantly associated with NM. Our study demonstrated that neonates who did not breastfeed exclusively were at a higher risk of death as compared to their exclusively breastfed counterparts. This finding is supported by studies conducted in Northern Ethiopia [12], and Bangladesh [37]. A study conducted in other SSA countries revealed that if breastfeeding was initiated within the first day of birth, the risk of NM reduced by 16% and could be reduced by 22% if it is initiated within one hour [38]. It is well known that the first milk (colostrum) produced by the mother has the benefit of reducing diseases like respiratory infections and otitis media, which ultimately contributes to the survival of neonates [39]. Evidence also suggested that breastfeeding reduces the risk of NM mortality in related to neonatal infections (i.e., sepsis, pneumonia, tetanus, and diarrhea) [40].

Moreover, this study found that neonates admitted to the NICU due to respiratory distress syndrome were at higher risk of death as compared to neonates admitted due to other causes. This finding is consistent with a study done in India showing that prematurity with respiratory distress syndrome and perinatal asphyxia were the two most common causes of NM [41]. The possible explanation for the high mortality of neonates due to respiratory distress syndrome could be due to the vulnerability of the study participants. For example, in our study, from all neonates admitted due to respiratory distress syndrome, about 49% were premature. Different literature documented that respiratory distress syndrome is the most common cause of death among premature neonates [42, 43].

Finally, this study showed that neonates whose first minute Apgar score classified as severe were at a higher risk of death as compared to those whose first minute Apgar was not classified as severe. This finding is consistent with a study conducted in Brazil, which showed a NM rate with the first minute Apgar score < 4 among 1000–1500 g weight group was threefold, and 35-fold ≥3000 g group [44].

### Limitations and strengths of the study

The main strength of this study is it was conducted prospectively. Therefore, we were able to include a range of sociodemographic, obstetric and neonatal variables, which were very important to determine NM. Despite these strengths, this study has a number of limitations. Firstly, the study was conducted at a hospital, therefore neonates delivered at home and died at home could be missed. Moreover there was a high ENM before initiating exclusive breastfeeding; consequently the true association between NM and breastfeeding could be overestimated. Furthermore, in this study, the impact of providers’ training, supplies, equipment, and hospital service contexts have not been explored.

## Conclusion

In this study, we found a high rate of ENM. Unemployed mothers, not attending ANC, not initiating exclusive breastfeeding, neonates admitted due to respiratory distress syndrome, and first minute Apgar score classified as severe were factors significantly predictive of increased the risk of NM. Therefore, based on our findings, we strongly recommend that special emphasis shall be given to neonates admitted during the early neonatal period. Training about the management of neonates with respiratory distress syndrome should be given to nurses and physicians working in the NICU of Debre Markos Referral Hospital. Furthermore, education about the importance of ANC and exclusive breastfeeding shall be given for the mothers during ANC care as well as postnatal care. Besides, neonates admitted due to early neonatal infections, asphyxia, and prematurity should get a special attention because 61.2% of the neonatal deaths were associated with the above three conditions. Lastly, further research is needed to explore the impact of provider training, supplies, equipment, and context.

## Abbreviations

ANC: Antenatal Care
CI: Confidence Interval
EDHS: Ethiopia Demographic and Health Survey
ENM: Early Neonatal Mortality
KM: Kilometer
LMICs: Low and Middle Income Countries
LNM: Late Neonatal Mortality
MDGs: Millennium Development Goals
NICU: Neonatal Intensive Care Unit
NM: Neonatal Mortality
SSA: Sub-Saharan Africa
WHO: World Health Organization
